# Post-acute sequelae of COVID-19 (PASC) are associated with distinct clinical phenotypes two years after SARS-CoV-2 infection: A data-driven approach

**DOI:** 10.1371/journal.pone.0359844

**Published:** 2026-10-15

**Authors:** Sander C. J. Verfaillie, Anouk Verveen, Denise Visser, Pythia Nieuwkerk, Brent Appelman, Ronald Boellaard, Caroline M. van Heugten, Janneke Horn, Hanneke E. Hulst, Menno D. de Jong, Tanja A. Kuut, Tessa van der Maaden, Yvonne M. G. van Os, Maria Prins, Johanna M. A. Visser-Meily, Michele van Vugt, Cees C. van den Wijngaard, Bart N. M. van Berckel, Jos A. Bosch, Nelleke Tolboom, Hans Knoop

**Affiliations:** 1 Department of Medical Psychology, Amsterdam UMC, Location AMC, Amsterdam, Netherlands; 2 GGz inGeest Specialized Mental Health Care, Amsterdam, Netherlands; 3 Department of Psychiatry, Amsterdam UMC Location VUmc, Amsterdam, Netherlands; 4 Department of Infectious Diseases, Amsterdam Institute for Infection and Immunity, Amsterdam, Netherlands; 5 Amsterdam Public Health, Amsterdam, Netherlands; 6 Department of Radiology & Nuclear Medicine, Amsterdam UMC Location Vrije Universiteit Amsterdam, Amsterdam, Netherlands; 7 Department of Radiology and Nuclear Medicine, Division of Imaging and Oncology, University Medical Center Utrecht, Utrecht, Netherlands; 8 Center for Experimental and Molecular Medicine, Amsterdam UMC Location AMC, Amsterdam, Netherlands; 9 Department of Psychiatry and Neuropsychology, Maastricht University, Maastricht, Netherlands; 10 Department of Intensive Care, Amsterdam UMC Location AMC, Amsterdam, Netherlands; 11 Health, Medical and Neuropsychology Unit, Institute of Psychology Leiden University, Leiden, Netherlands; 12 Department of Medical Microbiology & Infection Prevention, Amsterdam UMC Location AMC, Amsterdam, Netherlands; 13 Center for Infectious Disease Control, National Institute for Public Health and the Environment, Bilthoven, Netherlands; 14 Department of Occupational Health, Human Resources, University Medical Center, Utrecht, Netherlands; 15 Department of Infectious Diseases, Amsterdam UMC Location AMC, Amsterdam, Netherlands; 16 Department of Infectious Diseases, Public Health Service of Amsterdam, Amsterdam, Netherlands; 17 Department of Rehabilitation, Physical Therapy Science & Sports, UMC Utrecht Brain Center, University Medical Center Utrecht, Utrecht, Netherlands; 18 Department of Internal Medicine, Amsterdam UMC Location AMC, Amsterdam, Netherlands; Shanghai Jiao Tong University School of Medicine, CHINA

## Abstract

It is important to determine whether the persistent cognitive and fatigue complaints, as part of post-acute sequelae of COVID-19 (PASC), are associated with underlying patterns and specific symptoms clusters. Therefore, the aim of the present study was to investigate symptom clusters and clinical phenotypes in PASC. We included n = 153 individuals (53 years old, 66% female, 2 years post SARS-CoV-2 infection) categorized into groups with (n = 88) and without (n = 65) PASC, defined by severe fatigue and concentration problems using validated cut-offs. Symptom clusters (i.e., component loadings and component scores) were identified using principal component analyses (PCA, eigenvalue[EV]>1.0), using clinical, somatic, coping and sleep factors, symptom severity, neuropsychological and physical tests (totalling 19 tests and questionnaires). We then used logistic regression techniques on (participant-level) component scores (standardized) to investigate associations with PASC. Finally, K-means clustering methods to identify clinical phenotypes. Analyses were adjusted for age, sex, education, nationality and time since infection. PCA identified 5 components, explaining 70% of the variance. We labelled these components as follows: (1) PEM, ME/CFS and sleep symptoms, coping factors (EV = 7.0, 36.9% variance), (2) information-processing speed (EV = 2.5, 13.3%), (3) executive functioning (EV = 1.5, 8.0%, (4) physical performance (EV = 1.2, 6.5%), (5) somatic comorbidities and hospital admission (EV = 1.0, 5.5%). Logistic regression showed that only the first component scores (Odds ratio [95% confidence interval] = 97.1 [16.5–570.3], p < 0.001,), and none of the others (all p > 0.05), associated with PASC. Cluster analysis revealed two distinct clinical phenotypes among individuals with severe chronic fatigue: one marked by physical impairment and the other by diminished cognitive performance. Consistent with existing literature, we confirmed that PASC is a heterogeneous syndrome marked by extensive, interrelated symptoms. Notably, we identified specific cognitive and physical clinical phenotypes. These findings are important for future targeted diagnosis and treatment.

## Introduction

A large number of individuals have developed persistent complaints after initial SARS-CoV-2 infection, commonly referred to as post-acute sequelae of COVID-19 or PASC. Severe fatigue and cognitive complaints, which often co-occur, frequently persist for months after infection, even in the absence of evident neurological pathology or severe initial respiratory disease [1–4]. Recent studies estimate that 13–45% of COVID-19 survivors, regardless of hospitalisation status, report at least one unresolved symptom approximately 3–4 months after infection [2,5]. A substantial proportion of individuals with PASC report diminished participation in professional and daily activities and experience impaired quality of life [6,7].

PASC is a heterogeneous syndrome marked by extensive, interrelated symptoms, with evidence suggesting distinct clinical phenotypes. Clustering studies revealed clusters with low/high symptom burden [8], as well as mental and physical impairment [9–11] or distinct somatic symptom clusters including neurological, cardiorespiratory and heterogeneous symptoms [12]. However, these studies have investigated groups with relatively short PASC durations, ranging from 12 weeks [9,12] to 7 months [10], and long-term phenotypes remain unclear. In addition, much of the research has focused on specific subjective complaints and has not integrated subjective and objective measures in clustering methods. Various somatic, clinical, physical, cognitive, and psychological factors might simultaneously contribute to PASC and these could be intercorrelated. However, it is unclear whether there are distinct clinical phenotypes linked to PASC after approximately 2 years, particularly in individuals with persistent fatigue and difficulty concentrating.

Studying clinical characteristics is particularly important to unravel potential symptom clusters, as these may improve PASC diagnosis and may provide a more targeted treatment window for future therapies [11,12]. Therefore, the aim of this study is to investigate identify symptom clusters and clinical phenotypes associated with PASC in individuals with and without persistent fatigue and difficulty concentrating 2 years after initial SARS-CoV-2 infection.

## Materials and methods

### Participants

We included 153 individuals with and without persistent fatigue and difficulty concentrating, drawn from the data from the VeCosCO study, a case-control study in participants with a previous SARS-CoV-2 infection. To ensure compatibility with the principal component analysis (PCA), only participants with complete clinical, somatic, behavioural, and cognitive data were included. A detailed study protocol was published previously [13].

In summary, individuals with and without persistent fatigue and difficulty concentrating were included at least three months after their first confirmed SARS-CoV-2 diagnosis, recruited from existing prospective COVID-19 cohorts (study acronyms: ReCOVer, NeNeSCo, RECoVERED, LongCOVID) and were included between 10/05/2022 and 13/09/2023. Groups with and without persistent complaints were defined by the Checklist Individual Strength (CIS) questionnaire subscales for fatigue severity and concentration problems [13] and using validated cutoffs (subscale fatigue ≥35 and concentration ≥18) with a high sensitivity and specificity in detecting these symptoms [12,13]. Exclusion criteria were 1) a current psychiatric or somatic condition that could explain the presence of fatigue and/or concentration problems; 2) having severe fatigue or cognitive complaints prior to COVID-19; 3) insufficient Dutch language proficiency, or 4) re-infection with SARS-CoV-2 within 3 months prior to study inclusion. The authors assert that all procedures contributing to this work comply with the ethical standards of the relevant national and institutional committees on human experimentation and with the Helsinki Declaration of 1975, as revised in 2013. The study protocol (NL79575.018.21) was approved by the medical ethical review board of the Amsterdam University Medical Centers (Amsterdam, The Netherlands). Written informed consent was obtained from all subjects. Because of substantial missing data (range: 2–9% missing values; FES [4%], DSQ-SF [2%], PSQ [8%], ISI [3%], JFCS [2%], CBRSQ all or nothing [9%], CBRSQ damage beliefs [9%], CBRSQ avoidance [9%], SES28 [4%], HADS-D [4%], MRCSS [7%], SPPB [6%]), n = 77 individuals were not included in analyses. We chose to run analyses on original data because 1) the degree of missing data 2) uncertainties regarding clinical characteristics in PASC. We repeated analyses on imputed data the full dataset, presented in the Results section and supplemental data (S1 Table in S1 File).

### Clinical, somatic, sleep, cognitive-behavioural and neuropsychological measures

The following clinical, somatic, physical and chronic fatigue measures were used: Medical Research Council Sum Score (MRCSS), DePaul Symptom Questionnaire SF (DPSQ-SF, average score), fatigue and energy scale (FES), Short Physical Performance Battery (SPPB), medical comorbidities, hospital admission during initial SARS-CoV-2 infection (yes/no). Because there is a large symptom overlap between PASC and ME/CFS, we used the DPSQ-SF to measure chronic fatigue symptom severity (averaged severity*frequency scores of 14 items). The MRCSS measures skeletal muscle function (lower score reflects worse muscle strength). Lower extremity function was assessed by measuring walking speed, balance and leg strength with the Short Physical Performance Battery (SPPB: lower score reflects worse performance and mobility). Total number of the following self-reported comorbidities (before the first SARS-CoV-2 diagnosis) were calculated and included: cardiovascular disease (including hypertension), chronic pulmonary disease (including asthma), diabetes, neurological disease, haematological disease, rheumatic disease, thyroid disorders, renal disease, liver disease, cancer, and psychiatric illness. The FES is used to assess post-exertional exacerbation of fatigue and was administered after the neuropsychological assessment for a reflection of post-exertional malaise (PEM) due to cognitive exertion.

Based on earlier neuropsychological findings in individuals with and without PASC [14], we used the following neuropsychological tests: information processing speed (Stroop 1, Stroop 2, D2-test concentration performance) and executive functioning (Trialmaking Test B and B/A ratio). Lower scores reflect worse performance, and analyses were performed on age-, sex- and education-adjusted data (i.e., T-scores).

Sleep problems were assessed using the Insomnia Severity Index (ISI) and the Pittsburgh Sleep Questionnaire (PSQ). Depressive symptoms were measured using the Hospital Anxiety and Depression Scale (HADS) depression subscale.

The following coping factors were measured: responses to symptoms were assessed by three subscales of the 16-item Cognitive and Behavioural Responses to Symptoms Questionnaire (CBRSQ; subscales avoidance, damage and all-or-nothing beliefs), self-efficacy concerning fatigue was measured Self-Efficacy Scale (SES), and catastrophising of fatigue was assessed Jacobson-Fatigue Catastrophising Scale (JFCS). For all variables higher scores reflect more problematic or dysfunctional cognitive-behavioural responses to symptoms, except for SES 28. We used continuous total scores for each questionnaire for statistical analyses.

The following variables were used for descriptive purposes, degree of functional impairment or for statistical modelling and adjustments: age, sex, education (Verhage scale), nationality (Dutch origin yes/no), time since 1^st^ infection (in years), cognitive failure questionnaire [CFQ], premorbid psychiatric history (yes/no), and Post-COVID-19 Functional Status Scale (PCFS) [15].

### Statistical analyses

Data were entered into a secured electronic case report form (Castor EDC). Statistical analyses were performed using SPSS (v28). To investigate symptom clusters associated with persistent complaints we performed a PCA on clinical, somatic, PEM, ME/CFS, sleep, coping, physical functioning and neuropsychological variables (on cases with complete data) to derive data-driven component loadings and scores of PASC characteristics. Component selection was based upon the following retention diagnostics: eigenvalues >1, screeplot interpretation and percentage explained variance. Because cases with complete data were selected we tested for potential attrition using parametric and non-parametric tests. Subsequently, component loadings were interpreted and participant-level component scores were extracted and (Eigenvalues [EV] >1) using a Varimax rotation, to obtain components that are uncorrelated and, therefore, better interpretable. A component score is standardized (Mean ≈0, SD ≈ 1), reflecting a participant’s position on a component dimension derived from multiple variables, and is a degree of expression of the symptom cluster represented by the component. As an additional sensitivity analyses we re-ran PCA on a imputed dataset (n = 5 imputations) to test the robustness of our identified components. For descriptive and exploratory purposes we additionally performed Spearman rank correlation between component scores and socio-demographic variables (age, sex, education, nationality).

In a second set of analyses, we performed logistic regressions to investigate whether persistent complaints of fatigue and cognitive problems (binary independent variable) were associated with specific component scores (dependent variables), with and without adjustments for age, sex, education, nationality and time since infection. In sensitivity analyses, we performed linear regression analyses and using PCFS (continuous score) as a reflection of Post-COVID-19 functional impairment (instead of CIS-based groups). Finally, we clustered individuals based on the participant-level PCA components scores using K-means clustering techniques to identify clinical phenotypes. We used the elbow method and within-cluster sum of squares to determine the number of clusters, with a minimum of 3 clusters to avoid clustering of patient groups (with versus without persistent complaints). We finally used χ^2^ tests to investigate between-group cluster distributions. P-values <0.05 were considered statistically significant and we additionally applied a false-discovery rate (FDR) correction for multiple comparisons, if applicable.

## Results

Table 1 presents the socio-demographic and clinical characteristics of our sample and data used for principal component analyses. Individuals (n = 153) had a mean age of (mean± standard deviation) 53 ± 14 years old, 66% were female, with higher average levels of education. Fatigue and concentration complaints ranged from absent to severe (CIS subscale fatigue 33.7 ± 15.2, CIS subscale concentration 19.4 ± 8.6), and no to mild degree of depressive symptoms (HADS depression subscale 4.4 ± 4.0). Individuals with persistent complaints showed most frequently moderate functional limitations according to the PCFS (45% scored 3 [out of 4]), whereas individuals without persistent complaints showed no substantial functional limitation (78% scored 0). In addition, compared to individuals without complaints, individuals with persistent complaints were more often female (p < 0.001) and relatively younger (age 60 ± 15 versus 49 ± 12 years old, p < 0.001), but showed no differences with respect to education levels, hospital admission rates and time since infection (all p > 0.05).

**Table 1:** Socio-demographic and clinical characteristics.

	Total group	No PASC	PASC
	N = 153	N = 65	N = 88
Female sex	101 (66%)	55 (36%)	69 (78%)***
Age, years	53.4 (14.1)	59.8 (15.1)	48.8 (11.9)***
Education (Verhage Classification [range 1–7]) ^a^	5.9 (0.8)	6.1 (1.0)	5.9 (0.8)
Nationality (Dutch yes/no)	136 (90%)	56 (88%)	80 (91%)
			
**Somatic and clinical variables**			
Number of comorbidities^c^	0.7 (1.0)	0.7 (0.9)	0.8 (1.0)
Psychiatric comorbidity before COVID-19	9 (6%)	4 (6%)	5 (6%)
ApoE-e4 genotype, % e4 carriers	41/144 (28%)	21/61 (34%)	20/83 (24%)
Years since first SARS-CoV-2 infection	1.8 (0.6)	1.8 (0.6)	1.9 (0.6)
SARS-CoV-2 reinfection	54 (35%)	19 (30%)	35 (39%)
Unvaccinated at the time of 1^st^ infection	111 (73%)	43 (66%)	68 (77%)
Hospital admission for COVID-19	45 (29%)	23 (36%)	22 (25%)
Body Mass Index (BMI, kg/m2)	26.4 (5.0)	26.1 (4.9)	26.7 (5.2)
			
**ME/CFS and COVID-19 specific questionnaires**			
Post-COVID-19 Functional Status ^a^	1.6 (1.3)	0.4 (0.8)	2.4 (0.8)***
DePaul Symptom Questionnaire (DSQ-SF)	2.1 (1.9)	0.4 (0.4)	3.3 (1.8)***
Fatigue and Energy Scale (FES)	3.5 (2.8)	0.8 (1.1)	5.4 (2.2)***
			
**Affective variables**			
Hospital Anxiety Depression Scale (HADS) depression subscale	4.4 (4.0)	1.6 (2.2)	6.7 (3.8)***
			
**Neuropsychological assessment**			
Cognitive Failure Questionnaire (total)	39.7 (17.1)	25.7 (11.3)	49.6 (14.1)***
Test of Memory Malingering (trial 1)	47.9 (3.1)	49.6 (1.1)	47.0 (4.0)***
Stroop 1: word reading^d^	47.7 (12.4)	50.1 (10.4)	46.9 (13.8)
Stroop 2: colour naming^d^	47.5 (12.5)	51.4 (9.1)	45.9 (14.1)**
D2 test: continuous performance^d^	52.4 (10.5)	55.1 (10.5)	50.4 (10.2)**
Trialmaking Test version B	52.9 (10.8)	56.1 (9.6)	50.7 (11.1)**
Trialmaking Test B/A ratio	50.4 (9.8)	53.3 (8.2)	48.3 (10.1)**
			
**Physical variables**			
Medical Research Council (MRC)	28.7 (1.8)	29.1 (1.5)	28.4 (2.0)*
Short Physical Performance Battery (SPPB)	10.8 (1.6)	11.1 (1.3)	10.4 (1.9)**
			
**Cognitive-behavioural factors**			
Jacobson-fatigue catastrophizing scale (JCS)	16.7 (6.5)	11.4 (2.3)	20.7 (6.2)***
Self-efficacy Scale 28 (SES)	19.9 (3.7)	22.0 (3.9)	18.2 (2.7)***
Cognitive and Behavioural Responses to Symptoms Questionnaire (CBRSQ 16-item)			
- Fear avoidance - Damage beliefs - All-or-nothing behaviour	9.9 (5.6) 7.9 (3.7) 7.8 (4.6)	5.0 (3.4) 5.8 (3.2) 4.8 (3.4)	13.6 (4.4)*** 9.5 (3.4)*** 9.8 (4.2)***
			
**Sleep problems**			
Insomnia Sleep Index (ISI)	7.9 (5.7)	3.8 (3.9)	10.5 (5.3)***
Pittsburgh Sleep Questionnaire (PSQ)	6.8 (4.0)	4.1 (2.7)	8.7 (3.9)***

Because only cases with complete data (n = 153) were included for the primary analyses, we tested for potential attrition compared to the complete dataset of the VeCosCO study, and these analyses showed no attrition bias for age, sex, education, nationality, time since infection (supplemental data S1 Table in S1 File) between cases with available and incomplete data.

Continuous variables presented as mean (Standard deviation) or as frequency (%).^a^ Verhage educational classification system ranges from 1 (primary school) to 7 (University – Master’s degree) ^b^ Post-Covid functional status is categorical (0, no functional impairment – 4 severe functional impairment) ^c^ comorbidities prior to the first SARS-CoV-2 diagnosis: cardiovascular disease (including hypertension), chronic pulmonary disease (including asthma), diabetes, neurological disease, haematological disease, rheumatic disease, thyroid disorders, renal disease, liver disease, cancer, and psychiatric illness. ^d^ Stroop tests are presented with T-scores (age, sex, education adjusted by means of normative data). * p < 0.05, ** p < 0.01, *** p < 0.001

### PCA-derived component loadings and scores

In our main analyses, across the entire sample and with the use of PCA, we identified 5 components with an EV > 1, explaining 69% of the variance (please see a screeplot in Fig 1A). We labelled these components as follows: (1) PEM, ME/CFS and sleep symptoms, coping factors (EV = 7.0, 36.9% variance), (2) information-processing speed (EV = 2.5, 13.3%), (3) executive functioning (EV = 1.5, 8.0%, (4) physical performance (EV = 1.2, 6.5%), (5) somatic comorbidities and hospital admission (EV = 1.0, 5.5%). Table 2 presents the factor loadings for each variable and per component. The majority of variables loaded on component 1 (factor loading >0.5): coping factors (JFCS, SES28, CBRSQ), sleep problems (ISI, PSQI), ME/CFS symptoms including post-exertional exacerbation of fatigue (FES, DSQ-SF) and depressive symptoms (HADS-D). Component 2 consisted of neuropsychological tests (Stroop naming, Stroop colour naming and D2 test continuous performance) reflecting Information processing speed, whereas component 3 consisted of tests (TMT-B and TMT B/A ratio) reflecting executive functioning. Skeletal and physical function (MRC and SPPB) loaded on component 4 and hospital admission and somatic comorbidity loaded on component 5.

**Table 2:** Factor loading for each variable per component.

Components	1	2	3	4	5
**Variables**					
Component 1					
FES	**.826**	−.146	−.064	−.0186	−.014
DSQ – short form	**.841**	−.206	−.135	.140	.058
PSQI	**.782**	−.253	−.275	.024	.093
ISI	**.786**	−.131	.236	.048	.084
JFCS	**.848**	−.063	−.171	−.094	−.051
CBRSQ (all or nothing)	**.610**	.146	−.302	−.015	−.062
CBRSQ (damage beliefs)	**.637**	.024	−.373	−.106	−.038
CBRSQ (avoidance)	**.737**	−.155	−.285	−.076	−.070
SES28	**−.6056**	.159	.071	−.144	−.135
HADS depression subscale	**0.753**	−.047	−.178	−.277	−.135
Component 2					
Stroop 1	−.094	**.855**	.029	.029	−.011
Stroop 2	−.144	**.825**	.114	.138	.027
D2 test continuous performance	−.151	**.686**	.227	.007	−.004
					
Component 3					
Trialmaking Test version B	−0.08	.570	**.683**	.012	−.105
Trialmaking Test B/A ratio	−.121	.339	**.819**	.013	−.164
					
Component 4					
MRC	−.182	−.039	.056	**.855**	.055
SPPB	−.143	0.311	−.258	**.770**	−.239
					
Component 5					
Hospital admission	−.103	−.100	−.044	.056	**.784**
Somatic comorbidities	.105	.112	.094	−.197	**.768**
					

Factor loadings were derived with a Varimax rotation and loading >0.5 are presented in **bold**

**Fig 1:**
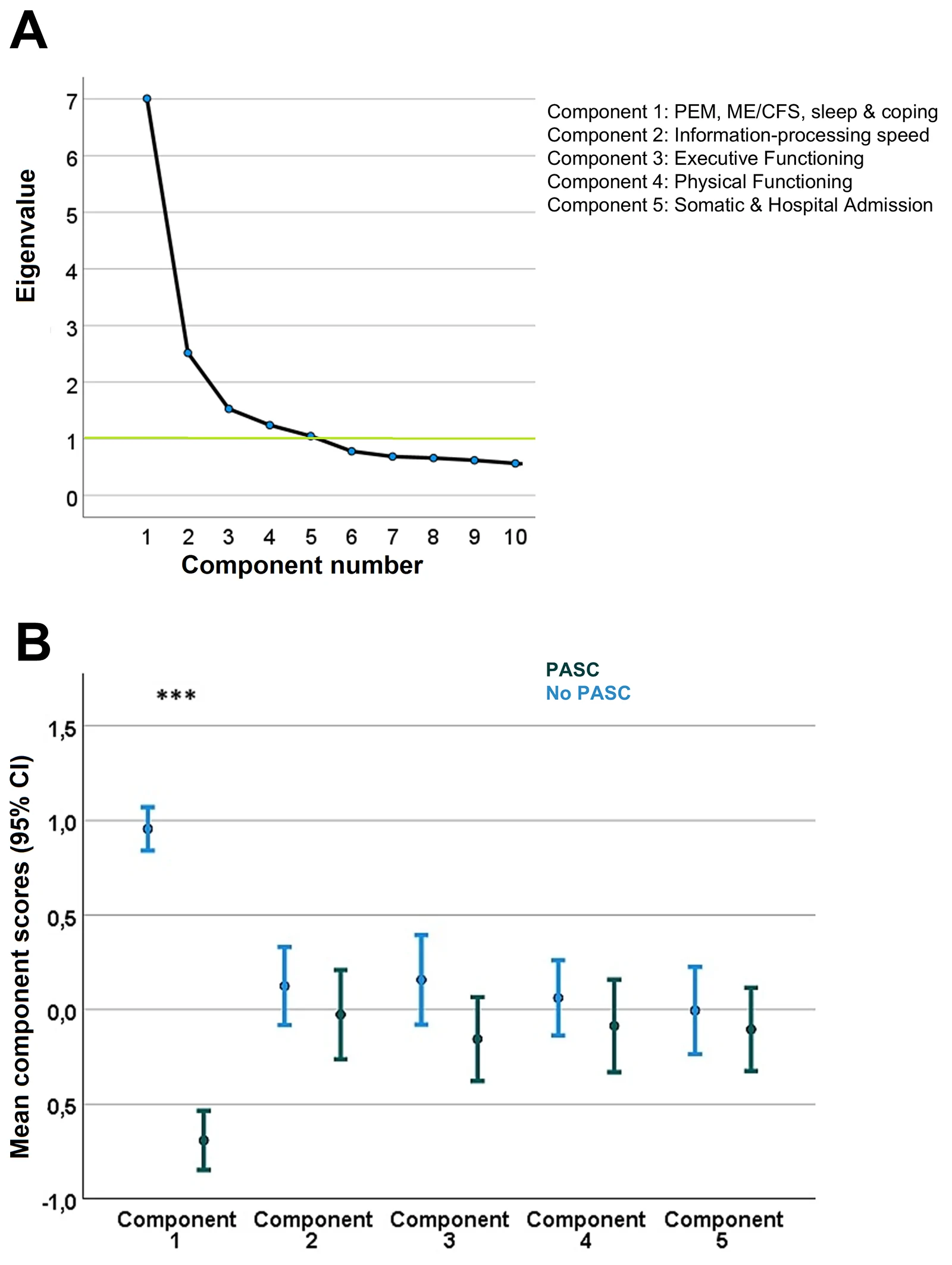
Panel A. Scree plot showing explained variance by each component. **Panel B**. shows participant-level component scores (with 95% confidence interval) for each component (Eigenvalue [EV]>1) split for individuals with and without persistent complaints. Lower scores on all components reflect worse functioning or more severe symptoms. Component 1 consisted of coping factors (Jacobson-Fatigue Catastrophising Scale [JFCS], Self-Efficacy Scale [SES28], 16-item Cognitive and Behavioural Responses to Symptoms Questionnaire [CBRSQ]), sleep problems (Insomnia Severity Index [ISI], Pittsburgh Sleep Quality Index [PSQI]), Myalgic encephalomyelitis/ chronic fatigue syndrome (ME/CFS) symptoms including post-exertional exacerbation of fatigue (Fatigue and Energy Scale [FES], DePaul Symptom Questionnaire [DSQ-SF]) and depressive symptoms (Hospital Anxiety Depression Scale – depression subscale [HADS-D]). Component 2 reflects information processing speed (Stroop naming, Stroop colour naming and D2 test continuous performance). Component 3 reflects, executive functioning (Trialmaking Test [TMT] versionB and TMT B/A ratio). Component 4 consisted of skeletal and physical function (Medical Research Council Sum Score [MRC] and Short Physical Performance Battery [SPPB]), and hospital admission and somatic comorbidity loaded on component 5. Statistical analyses on group differences were adjusted for age, sex, education, nationality and time since infection. * p < 0.05; ** p < 0.01; *** p < 0.001. EV = 1 is depicted with a green-coloured line in Panel A. Abbreviations: Confidence Interval, CI; Post-acute sequelae of COVID-19, PASC; Post-exertional Malaise, PEM.

Furthermore, because FES and DSQ-SF measure symptoms are closely relating to fatigue we re-ran PCA while excluding FES and DSQ-SF and the components remained unchanged, but explained less variance in on component 1 (33% versus 36%). Re-running the PCA on five imputed datasets yielded stable components (total explained variance range 64–74%), except for sleep problems (ISI, PSQI), which occasionally formed a distinct component.

### Associations between sociodemographic variables and PCA-derived components

For exploratory and descriptive purposes we performed Spearman rank correlations and these indicated correlations between female sex and worse factor scores on component 1 (Spearman’s rho [r_s_]= 0.41), whereas increasing age correlated with better scores on component 1 (r_s_ = 0.44), component 4 (r_s_ = 0.26) and component 5 (r_s_ = 0.33) (all p < 0.001). Higher education levels correlated with better scores on component 2 (r_s_ = 0.19) and worse scores on component 4 (r_s_ = −0.17) (both p < 0.05).

### Associations between persistent complaints and PCA-derived components

In a second set of analyses, we performed group logistic regression analyses on component scores (separately for all components) and likelihood for persistent complaints (i.e., PASC). Linear regression techniques showed that lower scores on component 1 ‘PEM, ME/CFS symptoms, sleep problems and coping factors’ were associated with increased likelihood of persistent complaints (Odds ratio [95% confidence interval] = 97.10 [16.52–570.34], p < 0.001), while adjusting for age, sex, education, nationality and time since infection and applying FDR corrections for multiple comparisons (see S2 Table in S1 File for associations with individual variables). There were no associations between persistent complaints and the other four components (all p > 0.05). In addition, if analyses were repeated while using the PCFS, as a reflection of severity functional impairment due to COVID-19, instead of (dichotomous) persistent fatigue and cognitive complaints, results remained comparable with component 1 showing the strongest association with functional impairments due to COVID19 (component 1: standardized beta = 0.64, p < 0.001 and component 4 standardized beta = −0.23, p = 0.38). No associations were found between PCFS and components 2, 3 and 5 (all p > 0.05).

### Clustering analyses

In a follow-up analyses we applied K-means clustering on participant-level component scores (of the initial PCA model) to identify clinical phenotypes (Fig 2; Table 3). Data was clustered in 3 subgroups with predominant: 1) physical impairment (n = 31 individuals [mean 52 years old, 83% females] of whom n = 24 [77%] with persistent complaints) 2) below average cognitive performance (n = 33 [53 years old, 71% females], of whom n = 28 [84%] with persistent complaints) and 3) relatively mild or no substantial problems (n = 89 [56 years old, 47% females], of whom n = 37 [42%] with persistent complaints). Cluster distribution was different between individuals with and without persistent complaints (p < 0.001).

**Table 3:** Frequency of clustering subgroups for individuals with and without PASC.

	No PASC	PASC	Total group
Cluster 1: physical impairment	7 (23%)	24 (77%)	31
Cluster 2: below average cognitive performance	5 (16%)	28 (84%)	33
Cluster 3: mild or no substantial problems	52 (58%)	37 (42%)	89

**Fig 2:**
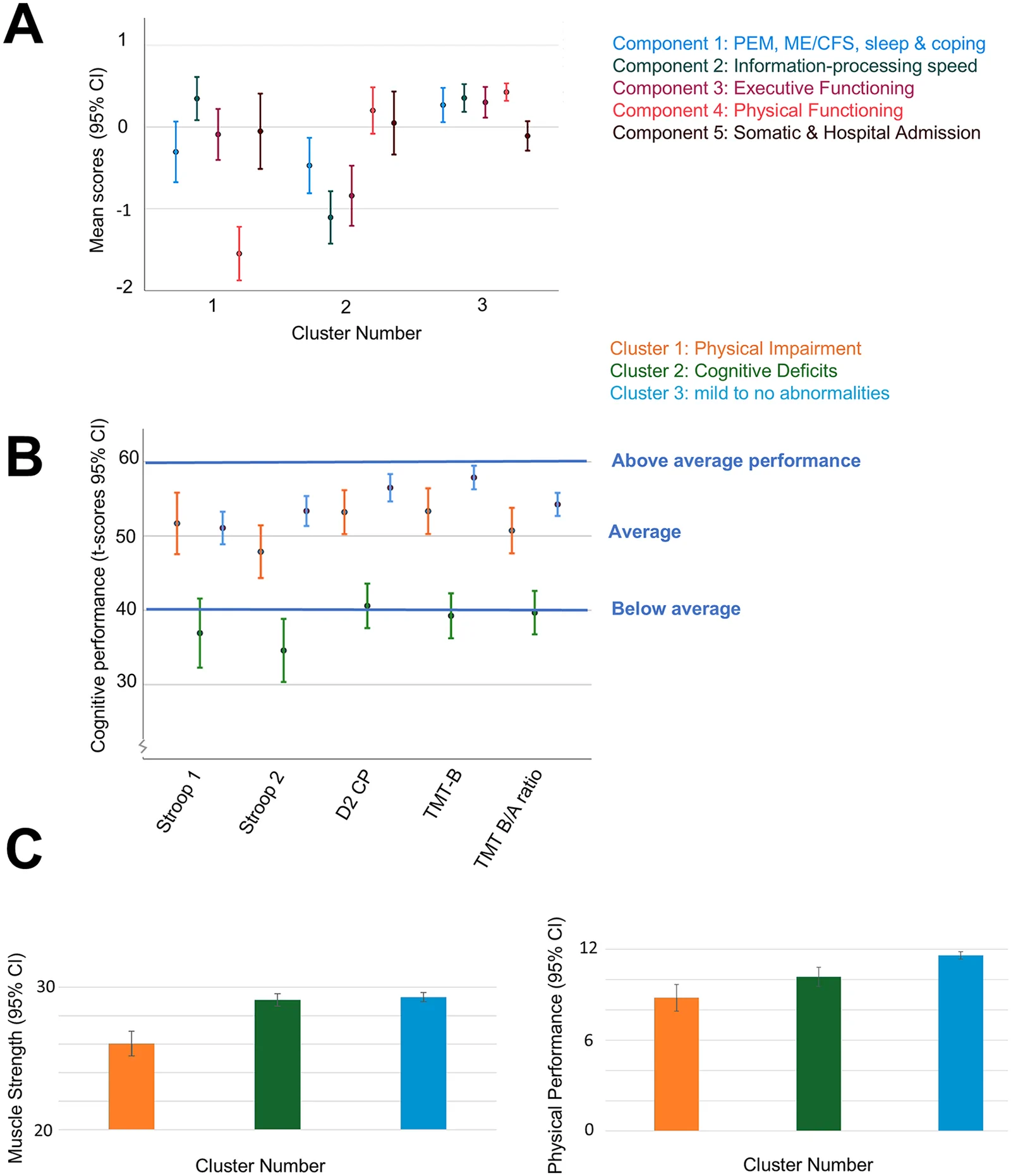
Panel A. Boxplots with component scores for each of the 5 components split for 3 K-means derived clusters demonstrating clinical profiles with either predominantly physical (cluster 1) or cognitive deficits (cluster 2) or relatively mild symptoms (cluster 3). Panel B. Neuropsychological test performance (expressed in T-scores) in the domains of information processing speed and executive functioning for each K-means derived clusters in individuals with PASC, with blue lines depicting below (−1SD compared to normative data) and above average (+1 SD) performance. Panel C. Muscle strength (left panel) and physical performance (right panel) for each K-means derived clusters in individuals with Post-acute sequelae of COVID-19 (PASC). Muscle strength (MRC) is calculated by grading (0-5) muscle strength of 6 muscle groups (0: no visible contraction, 30: normal strength), adapted protocol by Connolly et al., Critical Care (2013). Short Physical Performance Battery (SPPB) consists of three subtests (balance, gait speed and chair stand tests) with each section scored from 0 to 4 points for a total maximum score of 12 (score 5-8 reflects moderate mobility limitations). Abbreviations: Confidence Interval, CI; Myalgic encephalomyelitis/ chronic fatigue syndrome, ME/CFS; Post-exertional Malaise, PEM; Trialmaking Test, TMT.

## Discussion

In the present study we investigated symptom clusters and clinical phenotypes in individuals with and without persistent cognitive and fatigue complaints after COVID-19. In line with previous literature, we showed that PASC is a heterogeneous syndrome characterized by various interrelated symptoms, such as PEM, ME/CFS symptom severity, sleep problems, depressive symptoms and coping factors. We extend prior findings by showing that these symptoms primarily clustered into a single PASC-associated component, yet may also reflect distinct clinical phenotypes characterized by either more severe physical impairment or cognitive deficits. These results provide evidence for specific symptom clusters that may be targeted and mitigated with future (pharmacological or non-pharmacological) therapies.

The present study remains one of the few data-driven studies investigating clinical characteristics across various subjective and objective tests in individuals with and without PASC, two years after initial SARS-CoV-2 infection. Long-term studies have demonstrated that individuals with PASC exhibit substantially increases in, for example, depressive symptoms over time, suggesting a more dynamic disease course [16]. The interplay between long-term consequences, integrating psychological and medical factors, has not yet been thoroughly investigated. In the present study we investigated individuals from existing COVID-19 cohorts, mostly in the first year of pandemic, who were experiencing persistent complaints approximately 2 years after initial SARS-CoV-2 infection. This duration substantially exceeds the PASC definition of 3 months and may reflect a more chronic-stage of the disease rather than a post-acute stage. Using a PCA we reduced the number of dimensions of interrelated variables and symptoms incorporating self-reported ME/CFS, PEM, affective, sleep symptoms and coping factors, alongside objective measures of physical performance and cognitive performance and medical factors.

The largest proportion of variance was explained by a component dominated by subjective complaints and self-reported symptoms consisting of ME/CFS symptoms (e.g., PEM, unrefreshing sleep, myalgia, flu-like symptoms), sleep problems, depressive symptoms and aberrant cognitive and behavioural responses to illness symptoms. This component displays a large overlap of factors that are associated with ME/CFS [17,18] and can be explained by the observation that individuals with PASC often meet diagnostic criteria for ME/CFS [19]. More importantly, the aforementioned component was also strongly associated with persistent fatigue and cognitive complaints, whereas other components appeared unrelated. Of note, the primary component mainly involved subjective and self-reported symptoms whereas variables in other components used different assessment methods. In addition, because the first principal component is, by definition, the linear combination of variables that maximizes explained variance, it is also expected *a priori* that this component will show the strongest association with PASC status if group-related symptom burden constitutes a dominant source of variability. Therefore, we cannot rule out that components were influenced by methodological characteristics rather than content. Nevertheless, this component continued to show robust results in additional sensitivity analyses (including multiple imputations) and aligns with commonly reported subjective complaints related to PASC.

Using clustering methods, we identified two distinct PASC subtypes: one dominated by (mild) cognitive deficits, and another by physical impairment, both marked by elevated chronic fatigue-like symptoms as the primary feature. These two clusters mainly consisted of individuals with persistent complaints, and further supports evidence for distinct PASC clinical phenotypes. Subjective cognitive complaints are part of the post-COVID syndrome definition [20,21], and previous neuropsychological studies using clustering methods have shown that PASC is associated with specific objective cognitive profiles [22,23]. We extend these findings by providing evidence for specific clinical profiles of objective cognitive deficits together with other clinical symptoms in PASC using various subjective and objective physical and psychological tests, in addition to a neuropsychological assessment.

Interestingly, component loadings and scores consisting of somatic and physical factors as well as cognitive performance were not linked to persistent complaints if these were not accompanied with chronic fatigue symptoms. Discrepancies between subjective and objective measures, for example with respect to cognitive performance, have been demonstrated previously in PASC [14], as well as in other post-acute infectious syndromes like the Q fever fatigue syndrome [24]. An explanation could be that we have used insensitive neuropsychological tests for PASC or that the degree objective deficits related to PASC are generally small with negligible changes over time [25]. While relatively simple reaction times tend to be lower in PASC [26], a large study in a community sample, showed that individuals with persistent complaints after COVID-19 have only relatively small cognitive deficits (−0.33 to −0.20 SD) in the domains of memory, reasoning, and executive functioning [27]. In the present study, we demonstrate that at least one specific subgroup shows below-average information-processing and executive functioning.

The cross-sectional design obviously precludes causal inferences. Future longitudinal studies should further investigate cognitive dysfunctioning, post-exertional malaise, sleep problems and cognitive-behavioral responses to symptoms as they may each provide a potential treatment window (pharmacological or non-pharmacological) to alleviate persistent symptoms. A recent review found that cognitive-behavioral therapy and physical/mental health rehabilitation likely improve PASC symptoms, though the underlying pathophysiological mechanisms remain unclear [28].

In line with literature, the present study corroborates female sex as an important risk factor for PASC, because females showed on average higher scores on the primary component which consisted of typical PASC symptoms. However, we could not find evidence for other PASC-related risk factors, such as the presence of somatic comorbidity or hospital admission (acute phase) to be associated with persistent fatigue and cognitive complaints [10,29]. Furthermore, in contrast to the Post-Hospitalisation COVID-19 Study [10] we showed that physical performance (i.e., SPPB, component 2) was not exclusively linked to persistent complaints, which could also be explained by the fact that in the present study mainly non-hospitalised individuals were included.

A strength of the present study is that we included richly (clinically) phenotyped individuals with both subjective and objective measures with a 2 years after an initial SARS-CoV-2 infection. One advantage of this extended interval is that it allowed us to examine individuals who may be in a chronic phase of the condition. However, a limitation is that we cannot definitively rule out the influence of other factors, such as reinfections or social influences, on the course of PASC. This study also has other limitations. First, we have examined a relatively small size to explore a large set of factors and we excluded participants with extensive missing data which may have influenced our findings. Notwithstanding, component 1 appeared robust across various statistical analyses. To test for risk of attrition we compared demographic data between groups with and without missing data and we found no differences which suggests a low risk of bias or attrition (supplemental data). Second, (neuro)biological measurements were not taken into account. While the last years the number of somatic factors that have been studied as potentially contributing to PASC are substantial, it has not yet led to a valid and reliable (medical) diagnostic measurement and is beyond the scope of the present study. On the other hand, a recent study integrating biological, psychological, and social factors found that objective neurocognitive measures (e.g., MRI, neuropsychological assessment) were not associated with symptoms, but rather with psychological and social factors [30]. Third, PASC key symptoms like PEM and orthostatic intolerance were measured using subjective self-reports instead of thorough experimental testing such as the submaximal bicycle exercise stress test to investigate PEM. So far it has not been investigated whether experimental tests are better than subjective reports in PASC, but experimental tests may measure these phenomena more accurately and potentially could explain more (unique) variance. Finally, while we measured the number of comorbidities, we have insufficient data to explore individual effects of specific comorbidities and, therefore, their potential relevance to PASC.

In conclusion, we confirmed that PASC is a heterogeneous syndrome marked by extensive, interrelated symptoms. Notably, we identified specific cognitive and physical symptom clusters, that may reflect clinical phenotypes in individuals with a high burden of chronic fatigue. These findings are important for future targeted diagnosis and treatment. Our work adds to the emerging evidence that PASC might have distinct symptoms patterns, possibly linking to differing pathophysiological mechanisms, which are still poorly understood. Longitudinal studies are necessary to elucidate causal relationships between pathophysiological mechanisms, clinical phenotypes and symptoms.

## Supporting information

S1 File Supplemental materials Table S1 and Table S2. (DOCX)
