# Supplementary material for: Post-acute sequelae of COVID-19 (PASC) are associated with distinct clinical phenotypes two years after SARS-CoV-2 infection: A data-driven approach
Source: PLoS One. 2026 Oct 15;21(10):e0359844. doi: 10.1371/journal.pone.0359844 (PMC13671525; doi:10.1371/journal.pone.0359844)
Supplement: Supplementary file 1 [file pone.0359844.s001.docx]

**Supplemental data**

| **Sociodemographic variables** | **Rest of the cohort (n=77)** | **Present study**  **(n=153)** |
| --- | --- | --- |
| Time since infection (in years) | 1.98+0.59 | 1.86+0.61 |
| Dutch origin (yes/no) | 84% | 90% |
| Age (in years) | 55.92+12.91 | 53.38+14.36 |
| Education (Verhage classification 1-7) | 5.94+0.80 | 5.93+0.86 |
| Sex (% female) | 56% | 60% |
| CIS questionnaire (total score) | 77+34 | 77+32 |

**Table S1** Comparisons of means+SD for sociodemographic data for the entire cohort and sample used for the present study. Rest of the cohort consists of participants with missing data on any of the variables which were used for the principal component analyses (PCA)

|  | **Odd ratios (95% CI)** |
| --- | --- |
| **Variables Component 1** |  |
| Component 1 |  |
| FES | 11.5 (4.7 - 28.3) |
| DSQ – short form | 43.0 (9.2 - 200.1) |
| PSQI | 1.6 (1.3 – 2.0) |
| ISI | 1.4 (1.2- 1.6) |
| JFCS | 1.8 (1.5 - 2.3) |
| CBRSQ (all or nothing) | 1.3 (1.2 - 1.5) |
| CBRSQ (damage beliefs) | 1.4 (1.2 – 1.6) |
| CBRSQ (avoidance) | 1.6 (1,4 - 1.9) |
| SES28 | 0.7 (0,6 – 0.8)* |
| HADS depression subscale | 1.7 (1.4 – 2.1) |

Table S2. Odds ratios and 95% confidence intervals for each of the individual variables part of component 1. Odds ratios were adjusted for age, sex, education, nationality and time since infection (separate models). *lower scores mean increased likelihood of PASC
