# Supplementary material for: Post-acute sequelae of COVID-19 (PASC) are associated with distinct clinical phenotypes two years after SARS-CoV-2 infection: A data-driven approach
Source: PLoS One. 2026 Oct 15;21(10):e0359844. doi: 10.1371/journal.pone.0359844 (PMC13671525; doi:10.1371/journal.pone.0359844)
Supplement: Supplementary file 2 [file pone.0359844.s003.docx]

Manuscript: PONE-D-25-53636

“Post-acute sequelae of COVID-19 (PASC) are associated with distinct clinical phenotypes two years after SARS-CoV-2 infection: a data-driven approach”

Dear Dr. Kassegne,

We thank you and the two reviewers for your careful and constructive evaluation of our manuscript. We have revised the manuscript point-by-point in accordance with the comments raised, as detailed below. Reviewer and editor comments are reproduced in italics, followed by our response. All changes have been made in the revised manuscript and tracked-changes version.

We hope that the revised manuscript and our responses adequately address all concerns raised.

Yours sincerely,

On behalf of all authors,

Sander C.J. Verfaillie

Editorial comments:

Comments 1-4: We revised the manuscript with respect to PLOS ONE’s style requirements, with a validated ORCID ID for the corresponding author and Competing Interest statement in standard wording, and placed an ethics statement only in the Methods section, and included captions for our supporting information files at the end of our manuscript and update in-text citations accordingly.

*Comment 5. When completing the data availability statement of the submission form, you indicated that you will make your data available on acceptance. Please revise your statement, as your entire data will need to be made freely accessible if your manuscript is accepted for publication.*

We furthermore agree to make the data available and have inserted the following Data Availability Statement in the manuscript. Because legal or ethical restrictions prohibit public sharing of a data set, we indicated how others may obtain access to the data.

*“The Vecosco data are freely available to investigators under the conditions of a data sharing agreement. This will include group- and individual-level fully anonymized data. Requests for data should be made to prof.dr. H. Knoop.”*

*Comment 6. We note that you have included the phrase “data not shown” in your manuscript. PLOS does not permit references to inaccessible data.*

We agree and have removed this phrase from the manuscript (page 9)

*Conceptual clarity and inferential coherence of the PCA-based analyses

Because the PCA underpins the main Results and much of the Discussion, the following issues substantially affect the interpretability of the study’s core findings.
More broadly, there is a tension between the analytical method used (PCA) and the conceptual framing of the results. The Discussion repeatedly refers to latent factors, underlying constructs, and potential mechanistic or modifiable targets, which are assumptions inherent to factor-analytic or latent-variable models (e.g., EFA/CFA/SEM), but not to PCA. If the authors’ intention is to make claims about latent constructs underlying observed symptoms, a formal factor-analytic framework would be more appropriate. Alternatively, the Discussion should be revised to avoid interpretations that exceed what PCA can support.*

1. Our apologies for the confusion. We performed a PCA and corrected the terminology in the Abstract, Methods, Results and Discussion sections. Please see our more detailed response to the specific points below.

*TERMINOLOGY - Throughout the manuscript, the authors use closely related but not equivalent terminology interchangeably, which introduces important conceptual and methodological confusion between principal component analysis (PCA) and factor-analytic concepts. For example, the Methods state that “a PCA (…) was performed to derive data-driven components of PASC characteristics,” but shortly thereafter refer to “factor loadings” extracted after Varimax rotation and later describe the regression analyses as investigating associations with “specific latent factors.” PCA does not estimate latent variables and does not model measurement error or communalities; rather, it produces components, component loadings, and component scores as results of a variance-decomposition procedure. Terms such as “latent factors,” “factor loadings,” and “factor structure” are specific to factor-analytic models and are not interchangeable with PCA terminology.*

2. We thank the reviewer for raising this important point, and we apologize for the confusion caused by our imprecise use of terminology. We performed a PCA, and we have therefore revised the manuscript throughout (Abstract, Introduction, Methods, Results, Discussion, figures and tables) to consistently use PCA-appropriate terminology. Specifically, we have changed “latent factors” to “symptom clusters” (or “components,” as appropriate), “factor loadings” to “component loadings,” and have removed language implying latent constructs. The interpretation in the Discussion has been adjusted accordingly to reflect the descriptive, variance-decomposition nature of PCA rather than a latent-variable interpretation.

*COMPONENT RETENTION - Component retention appears to be based on the eigenvalue > 1 criterion. As this rule is known to be sensitive to the number of variables and to frequently overestimate the number of components, particularly in exploratory settings, the authors are encouraged to report additional retention diagnostics (e.g., scree plot inspection or parallel analysis) or to justify the robustness of the selected solution.*

3. We agree and have changed figure 1A, which includes a screeplot, to enhance interpretation. We furthermore provided eigenvalues of each components with corresponding percentages of explained variance in the manuscript, to support the component retention decision, and we have reported this in the Methods/Results (page 5 and 8) and figure 1A.

*LOADINGS / SCORES CONFUSION - The manuscript states that linear regression was performed “on factor loadings,” which is methodologically incorrect. Component (or factor) loadings are fixed properties of the PCA solution at the variable level and do not vary across individuals; as such, they cannot serve as dependent variables in regression analyses. The analyses appear instead to use component scores, which are subject-level quantities and are appropriate outcomes for regression. This distinction should be explicitly clarified and corrected throughout the manuscript.*

4. We agree and thank the reviewer for catching this error. We confirm that the regression analyses used component scores, not component loadings. We have corrected this terminology throughout the manuscript (Methods and Results section) to consistently and accurately distinguish loadings (variable-level) from scores (subject-level).

*CORRELATION VS CONFOUNDING - The manuscript reports Spearman correlations between sociodemographic variables (age, sex, education, ethnicity) and the PCA-derived components and describes this step as an assessment of “confounding effects.” However, confounding was already addressed in the subsequent multivariable regression models that adjusted for these same variables. Bivariate correlation does not assess confounding and instead only describes unadjusted associations. This analysis should therefore be described as exploratory or descriptive, or omitted.*

5. We agree and have rephrased this sentence (page 5 and 6) and to describe these bivariate correlations as exploratory/descriptive associations rather than as an assessment of confounding.

*INFERENTIAL TARGET / PC1 INEVITABILITY - More broadly, the objective and interpretability of the PCA-based regression analyses are unclear. Because the first principal component is, by definition, the linear combination of variables that maximizes explained variance, it is largely expected a priori that this component will show the strongest association with PASC status if group-related symptom burden constitutes a dominant source of variability. The subsequent finding that only the first unsupervised component distinguishes groups is therefore unsurprising and risks being over-interpreted as an inferential result rather than a descriptive property of the variance structure.*

6. We thank the reviewer for this insightful comment. We agree with this concern and have revised the Discussion (page 12) to explicitly acknowledge this limitation and to temper the interpretation of the first principal component.

*A clearer alignment between the research question and the analytical strategy would be achieved by either modeling PASC status directly—using PCA or factor scores purely as predictors in a logistic regression framework adjusted for covariates—or by testing multivariate group differences using PERMANOVA applied to the original symptom and test variables. In the latter case, an appropriate distance metric and scaling for the mixed variable types would allow formal inference on group differences in the full multivariate space, with a corresponding constrained ordination (e.g., redundancy analysis) providing an interpretable visualization. While PCA remains useful for descriptive dimension reduction and exploratory purposes (e.g., clustering), treating PCA components themselves as inferential outcomes obscures the group-comparison objective and risks over-interpreting variance-maximizing components.*

7. We thank the reviewer for this suggestion. In the revised version of the manuscript we ran a logistic regression with PCA scores as predictor and PASC as dependent variable, while adjusting for covariates. In the revised version we now present Odds ratios and 95% confidence intervals (page 6 and 10).

*Minor comment: choice of visualization in Figure 2B

Figure 2B presents cluster distributions using pie charts. With only three groups, this visualization offers limited interpretive value and makes quantitative comparison difficult. The same information could be more clearly conveyed using a simple table, a bar plot with proportions, or even brief textual reporting of frequencies and percentages. Replacing the pie charts with a more straightforward representation would improve clarity and readability.*

8. We agree and have replaced the pie charts in Figure 2B with a simple table reporting frequencies and percentages (table 3, page 11) for clearer quantitative comparison.

*Minor comment: clarity of Figure 2D. Figure 2D is intended to illustrate “muscle strength and physical performance,” but the units of measurement and the nature of the displayed uncertainty are not specified in the figure keys or caption. It is therefore unclear whether the panels show group means with error intervals (and, if so, whether these represent standard deviations, standard errors, or confidence intervals) or another summary measure.
For group-level summaries with associated uncertainty, alternative visualizations such as point–range plots (means with confidence intervals), boxplots, or violin plots would improve clarity and interpretability and allow readers to better assess the distribution of the underlying data. At a minimum, the figure caption and legend should explicitly state the scale, units, and definition of the error estimates used.*

9. In the revised manuscript we now provided information about the units of measurement and definition of the error in the legend. In both tests muscle strength and physical performance are assessed by a clinician-scientist and graded on a validated scale.

*Reviewer #2: This analysis included participants with and without persistent fatigue and difficulty concentrating. Using a data-driven approach, the manuscript noted heterogeneity, including symptoms such as PEM and sleep problems.*

*More details are necessary regarding inclusion criteria. As participants experienced persistent fatigue and difficulty concentrating, and these factors also appeared in the various factors examined. How are you distinguishing between inclusion criteria and outcomes of interest? For example, it's not surprising that the FES or DSQ would be highly correlated with being the PASC group.*

10. We agree that there is a substantial overlap between our inclusion criteria and variables that we examined. One explanation is that by design our aim was to investigate factors associated with PASC and we therefore selected PASC-sensitive tests. It is therefore not surprising that these highly correlated. Our inclusion criteria are based upon validated cut-offs (control versus patients) for substantial chronic fatigue and concentration problems which have also been applied in other syndromes/ diseases (e.g. ME/CFS, cancer). Notwithstanding to assess whether our inclusion criteria influenced we repeated our primary analyses with a more general Post-COVID Functional Scale (PCFS, continuous scale) and the results remained essentially unchanged (page 10). In another sensitivity analyses we re-ran PCA without FES and DSQ because of the high correlations between FES and DSQ and inclusion criteria. Results of these analyses indicated similar set of component, although explained somewhat less variance (3%) (page 9). Hence we feel that we that we have done justice to the data.

*The methods section notes that "only participants with complete clinical, somatic, behavioural, and cognitive data were included." How many participants were excluded because of this criteria? What pattern of missing observations was seen in the data? At minimum, a flowchart is necessary to describe included participants. But, could other methods have been used to include these participants? Also, on this topic, the methods section later states that imputation was used. It is unclear why 1) imputation is needed if only complete data was used, and 2) why imputation could not have been used all along with whatever values were missing? How was the number of imputations selected? How many participants were excluded because of this criteria? What pattern of missing observations was seen in the data? At minimum, a flowchart is necessary …*

11. In the total study we had data of n=230 individuals. Seventy-seven participants were excluded because they had missing data. Please also see response 12. We have compared demographics, fatigue and concentration severity of the complete data set with the one used for the present study and we did not see specific patterns of observations or evidence for attrition. Comparisons are presented in the supplemental data. In short, demographic and basic clinical data was comparable and there was no indication for a pattern of missing data.

To test if the missing data may give other findings we also performed imputations (n=5) on the entire dataset. PCA’s delivered essentially comparable factor loadings.

We did not use imputations all along because we found that many cases had substantial amount of missing data (2-9%) and preferred to work with the original data and subsequently test its robustness on imputed data.

In the revised manuscript we now provided the reader with the percentages of missing data for each variable, because we find this more informative than a flowchart (page 4).

*It is unclear why 1) imputation is needed if only complete data was used, and 2) why imputation could not have been used all along with whatever values were missing? How was the number of imputations selected?*

12. Apologies for the confusion, the primary analyses was performed on complete data (n=153). We have not used imputation all along because in some cases there was substantial amount of missing data. In addition, there are still many unknown factors that contribute to the clinical characteristics of PASC and we preferred to avoid influences to the data. However, to reassure that our results were not dependent of the cases with only available data, we repeated primary analyses with imputed data (default n=5). We have clarified the Methods to explain the rationale for using imputation in this context and how the number of imputations was selected.

*This analysis has a relatively small sample to explore a large number of factors. Were there any considerations related to sample size?*

13. We included all participants who have participated in the VeCosCO study, which is a prospective case-control study (Verveen et al., <https://doi.org/10.1136/bmjopen-2023-072611>). We agree that we examined a relatively sample size in relation to a large number of factors. We acknowledged this as a limitation in the Discussion (page 14).

*The approach using factor loadings and Eigenvalues along with a Varimax rotation loses the original scale of the included factors. How should the results be interpreted? For example, in Figure 1A, how should the Eigenvalues and values along the X-axis be interpreted? And in Figure 1B, what does it mean that component 1 has a factor loading around -0.7 for the PASC group and 0.95 for the no PASC group? In Figure 2A, what do 1, 2 and 3 mean on the X-axis? What is meant exactly by "latent factors"?*

14. We apologize for the confusion with respect to statistics. As noted above, we have used a PCA, and have therefore changed the formulation and interpretation throughout the manuscript from “latent factors” to “symptom clusters” (and component loadings/scores, as appropriate). We have also clarified in the Methods (page 5 and 6) and figure legends how the eigenvalues, component loadings, and component scores should be interpreted. In short, these are standardized scores (mean~0 and SD~1).

We furthermore provided information on the X-axis about the meaning of 1, 2 and 3 in Figure 2A. These represent K-means derived Cluster Numbers.

*Number of comorbidities was included as a factor. It is however possible that certain comorbidities could be more relevant in some clusters than others?*

15. We agree this is an interesting consideration. During the data collection we have not separately specified each comorbidity, so we are unable to investigate these effects unfortunately. We have added a comment on this point in the Discussion as a limitation (page 14)

*Dutch is nationality, rather than ethnicity.*

16. We have corrected this terminology throughout the manuscript.

*There is no data availability statement in the manuscript (despite a statement to the contrary on the first pages). Please include this information.*

17. We agree to make the data available and have inserted the following Data Availability Statement in the manuscript:

*“The Vecocsco data are available to investigators under the conditions of a data sharing agreement. This will include group- and individual-level fully anonymized data. Requests for data should be made to prof.dr. H. Knoop.”*
