# Supplementary material for: Post-acute sequelae of COVID-19 (PASC) are associated with distinct clinical phenotypes two years after SARS-CoV-2 infection: A data-driven approach
Source: PLoS One. 2026 Oct 15;21(10):e0359844. doi: 10.1371/journal.pone.0359844 (PMC13671525; doi:10.1371/journal.pone.0359844)
Supplement: Supplementary file 3 [file pone.0359844.s004.docx]

Manuscript: PONE-D-25-53636

“Post-acute sequelae of COVID-19 (PASC) are associated with distinct clinical phenotypes two years after SARS-CoV-2 infection: A data-driven approach”

Dear Dr. Kassegne,

We thank the reviewers for their careful and constructive evaluation of our manuscript in this second round. We are pleased to hear that they found the manuscript considerably strengthened during revision and now suitable for publication, pending minor editorial revisions. We have addressed all comments point-by-point, as detailed below. Reviewer comments are reproduced in *italics*, followed by our responses. All changes are reflected in the revised manuscript and the tracked-changes version.

We hope that the revised manuscript is suitable for publication.

Yours sincerely, on behalf of all authors,

Sander C.J. Verfaillie

Reviewer #3: The authors have addressed the reviewers' comments comprehensively and have substantially improved the manuscript. The statistical methodology is now much clearer, particularly regarding the appropriate interpretation of the PCA, the distinction between component loadings and component scores, and the revised regression analyses. The additional sensitivity analyses and the revised discussion strengthen the robustness and transparency of the findings.
The manuscript is well structured, the research question is clinically relevant, and the identification of different clinical phenotypes in PASC represents a valuable contribution to the field. The authors have also improved the presentation of the results by revising figures and tables, clarifying methodological details, and appropriately tempering conclusions where necessary.
I only have a few minor suggestions before publication:

1. A final proofreading for grammar and language would further improve readability, as a few minor stylistic issues remain.

*We have corrected grammar and language issues in the revised manuscript.*

2. Please ensure that PCA terminology (components, component loadings, component scores) is used consistently throughout the final manuscript.

*We have made several corrections to ensure consistent PCA terminology in the revised manuscript.*

3. Verify that all figure legends are fully self-contained, including definitions of abbreviations and statistical measures.

*We have revised the figure legends so that these are fully self-contained, including definitions of abbreviations.*
