# Supplementary material for: Post-acute sequelae of COVID-19 (PASC) are associated with distinct clinical phenotypes two years after SARS-CoV-2 infection: A data-driven approach
Source: PLoS One. 2026 Oct 15;21(10):e0359844. doi: 10.1371/journal.pone.0359844 (PMC13671525; doi:10.1371/journal.pone.0359844)
Supplement: Peer review history

# Peer review: Decision Letter 0

**DOI:** 10.1371/journal.pone.0359844.r001

**Authors:** Kokouvi Kassegne

29 Apr 2026

PONE-D-25-53636

Post-acute sequelae of COVID-19 (PASC) are associated with distinct clinical phenotypes two years after SARS-CoV-2 infection: a data-driven approach

PLOS One

Dear Dr. Verfaillie,

Thank you for submitting your manuscript to PLOS ONE. After careful consideration, we feel that it has merit but does not fully meet PLOS ONE’s publication criteria as it currently stands. Therefore, we invite you to submit a revised version of the manuscript that addresses the points raised during the review process.

Please submit your revised manuscript by Jun 13 2026 11:59PM. If you will need more time than this to complete your revisions, please reply to this message or contact the journal office at plosone@plos.org. When you're ready to submit your revision, log on to https://www.editorialmanager.com/pone/ and select the 'Submissions Needing Revision' folder to locate your manuscript file.

Please include the following items when submitting your revised manuscript:

- A letter that responds to each point raised by the academic editor and reviewer(s). You should upload this letter as a separate file labeled 'Response to Reviewers'.
- A marked-up copy of your manuscript that highlights changes made to the original version. You should upload this as a separate file labeled 'Revised Manuscript with Track Changes'.
- An unmarked version of your revised paper without tracked changes. You should upload this as a separate file labeled 'Manuscript'.

If you would like to make changes to your financial disclosure, please include your updated statement in your cover letter. Guidelines for resubmitting your figure files are available below the reviewer comments at the end of this letter.

If applicable, we recommend that you deposit your laboratory protocols in protocols.io to enhance the reproducibility of your results. Protocols.io assigns your protocol its own identifier (DOI) so that it can be cited independently in the future. For instructions see: https://journals.plos.org/plosone/s/submission-guidelines#loc-laboratory-protocols. Additionally, PLOS ONE offers an option for publishing peer-reviewed Lab Protocol articles, which describe protocols hosted on protocols.io. Read more information on sharing protocols at https://plos.org/protocols?utm_medium=editorial-email&utm_source=authorletters&utm_campaign=protocols.

As the corresponding author, your ORCID iD is verified in the submission system and will appear in the published article. PLOS supports the use of ORCID, and we encourage all coauthors to register for an ORCID iD and use it as well. Please encourage your coauthors to verify their ORCID iD within the submission system before final acceptance, as unverified ORCID iDs will not appear in the published article. Only the individual author can complete the verification step; PLOS staff cannot verify ORCID iDs on behalf of authors.

We look forward to receiving your revised manuscript.

Kind regards,

Kokouvi Kassegne

Academic Editor

PLOS One

Journal Requirements:

When submitting your revision, we need you to address these additional requirements.

1. Please ensure that your manuscript meets PLOS ONE's style requirements, including those for file naming. The PLOS ONE style templates can be found at

https://journals.plos.org/plosone/s/file?id=wjVg/PLOSOne_formatting_sample_main_body.pdf and

https://journals.plos.org/plosone/s/file?id=ba62/PLOSOne_formatting_sample_title_authors_affiliations.pdf

2. PLOS requires an ORCID iD for the corresponding author in Editorial Manager on papers submitted after December 6th, 2016. Please ensure that you have an ORCID iD and that it is validated in Editorial Manager. To do this, go to ‘Update my Information’ (in the upper left-hand corner of the main menu), and click on the Fetch/Validate link next to the ORCID field. This will take you to the ORCID site and allow you to create a new iD or authenticate a pre-existing iD in Editorial Manager.

3. Thank you for stating the following in your Competing Interests section:

"no competing interests"

Please complete your Competing Interests on the online submission form to state any Competing Interests. If you have no competing interests, please state "The authors have declared that no competing interests exist.", as detailed online in our guide for authors at http://journals.plos.org/plosone/s/submit-now

This information should be included in your cover letter; we will change the online submission form on your behalf.

4. Your ethics statement should only appear in the Methods section of your manuscript. If your ethics statement is written in any section besides the Methods, please delete it from any other section. "

5. When completing the data availability statement of the submission form, you indicated that you will make your data available on acceptance. We strongly recommend all authors decide on a data sharing plan before acceptance, as the process can be lengthy and hold up publication timelines. Please note that, though access restrictions are acceptable now, your entire data will need to be made freely accessible if your manuscript is accepted for publication. This policy applies to all data except where public deposition would breach compliance with the protocol approved by your research ethics board. If you are unable to adhere to our open data policy, please kindly revise your statement to explain your reasoning and we will seek the editor's input on an exemption. Please be assured that, once you have provided your new statement, the assessment of your exemption will not hold up the peer review process.

6.  We note that you have included the phrase “data not shown” in your manuscript. Unfortunately, this does not meet our data sharing requirements. PLOS does not permit references to inaccessible data. We require that authors provide all relevant data within the paper, Supporting Information files, or in an acceptable, public repository. Please add a citation to support this phrase or upload the data that corresponds with these findings to a stable repository (such as Figshare or Dryad) and provide and URLs, DOIs, or accession numbers that may be used to access these data. Or, if the data are not a core part of the research being presented in your study, we ask that you remove the phrase that refers to these data.

7. Please include captions for your Supporting Information files at the end of your manuscript, and update any in-text citations to match accordingly. Please see our Supporting Information guidelines for more information: http://journals.plos.org/plosone/s/supporting-information.

8. If the reviewer comments include a recommendation to cite specific previously published works, please review and evaluate these publications to determine whether they are relevant and should be cited. There is no requirement to cite these works unless the editor has indicated otherwise.

Additional Editor Comments:

Pease provide point-by-point response to the reviewers' comments and revise the manuscript accordingly to show where revisions are made.

[Note: HTML markup is below. Please do not edit.]

Reviewers' comments:

Reviewer's Responses to Questions

**Comments to the Author**

1. Is the manuscript technically sound, and do the data support the conclusions?

Reviewer #1: Partly

Reviewer #2: Yes

**********

2. Has the statistical analysis been performed appropriately and rigorously?

Reviewer #1: No

Reviewer #2: No

**********

3. Have the authors made all data underlying the findings in their manuscript fully available?

The PLOS Data policy

Reviewer #1: No

Reviewer #2: No

**********

4. Is the manuscript presented in an intelligible fashion and written in standard English?

Reviewer #1: Yes

Reviewer #2: Yes

**********

Reviewer #1: Major comment:

Conceptual clarity and inferential coherence of the PCA-based analyses

Because the PCA underpins the main Results and much of the Discussion, the following issues substantially affect the interpretability of the study’s core findings.

More broadly, there is a tension between the analytical method used (PCA) and the conceptual framing of the results. The Discussion repeatedly refers to latent factors, underlying constructs, and potential mechanistic or modifiable targets, which are assumptions inherent to factor-analytic or latent-variable models (e.g., EFA/CFA/SEM), but not to PCA. If the authors’ intention is to make claims about latent constructs underlying observed symptoms, a formal factor-analytic framework would be more appropriate. Alternatively, the Discussion should be revised to avoid interpretations that exceed what PCA can support.

Specific points:

TERMINOLOGY - Throughout the manuscript, the authors use closely related but not equivalent terminology interchangeably, which introduces important conceptual and methodological confusion between principal component analysis (PCA) and factor-analytic concepts. For example, the Methods state that “a PCA (…) was performed to derive data-driven components of PASC characteristics,” but shortly thereafter refer to “factor loadings” extracted after Varimax rotation and later describe the regression analyses as investigating associations with “specific latent factors.” PCA does not estimate latent variables and does not model measurement error or communalities; rather, it produces components, component loadings, and component scores as results of a variance-decomposition procedure. Terms such as “latent factors,” “factor loadings,” and “factor structure” are specific to factor-analytic models and are not interchangeable with PCA terminology.

COMPONENT RETENTION - Component retention appears to be based on the eigenvalue > 1 criterion. As this rule is known to be sensitive to the number of variables and to frequently overestimate the number of components, particularly in exploratory settings, the authors are encouraged to report additional retention diagnostics (e.g., scree plot inspection or parallel analysis) or to justify the robustness of the selected solution.

LOADINGS / SCORES CONFUSION - The manuscript states that linear regression was performed “on factor loadings,” which is methodologically incorrect. Component (or factor) loadings are fixed properties of the PCA solution at the variable level and do not vary across individuals; as such, they cannot serve as dependent variables in regression analyses. The analyses appear instead to use component scores, which are subject-level quantities and are appropriate outcomes for regression. This distinction should be explicitly clarified and corrected throughout the manuscript.

CORRELATION VS CONFOUNDING - The manuscript reports Spearman correlations between sociodemographic variables (age, sex, education, ethnicity) and the PCA-derived components and describes this step as an assessment of “confounding effects.” However, confounding was already addressed in the subsequent multivariable regression models that adjusted for these same variables. Bivariate correlation does not assess confounding and instead only describes unadjusted associations. This analysis should therefore be described as exploratory or descriptive, or omitted.

INFERENTIAL TARGET / PC1 INEVITABILITY - More broadly, the objective and interpretability of the PCA-based regression analyses are unclear. Because the first principal component is, by definition, the linear combination of variables that maximizes explained variance, it is largely expected a priori that this component will show the strongest association with PASC status if group-related symptom burden constitutes a dominant source of variability. The subsequent finding that only the first unsupervised component distinguishes groups is therefore unsurprising and risks being over-interpreted as an inferential result rather than a descriptive property of the variance structure.

A clearer alignment between the research question and the analytical strategy would be achieved by either modeling PASC status directly—using PCA or factor scores purely as predictors in a logistic regression framework adjusted for covariates—or by testing multivariate group differences using PERMANOVA applied to the original symptom and test variables. In the latter case, an appropriate distance metric and scaling for the mixed variable types would allow formal inference on group differences in the full multivariate space, with a corresponding constrained ordination (e.g., redundancy analysis) providing an interpretable visualization. While PCA remains useful for descriptive dimension reduction and exploratory purposes (e.g., clustering), treating PCA components themselves as inferential outcomes obscures the group-comparison objective and risks over-interpreting variance-maximizing components.

Minor comment: choice of visualization in Figure 2B

Figure 2B presents cluster distributions using pie charts. With only three groups, this visualization offers limited interpretive value and makes quantitative comparison difficult. The same information could be more clearly conveyed using a simple table, a bar plot with proportions, or even brief textual reporting of frequencies and percentages. Replacing the pie charts with a more straightforward representation would improve clarity and readability.

Minor comment: clarity of Figure 2D

Figure 2D is intended to illustrate “muscle strength and physical performance,” but the units of measurement and the nature of the displayed uncertainty are not specified in the figure keys or caption. It is therefore unclear whether the panels show group means with error intervals (and, if so, whether these represent standard deviations, standard errors, or confidence intervals) or another summary measure.

For group-level summaries with associated uncertainty, alternative visualizations such as point–range plots (means with confidence intervals), boxplots, or violin plots would improve clarity and interpretability and allow readers to better assess the distribution of the underlying data. At a minimum, the figure caption and legend should explicitly state the scale, units, and definition of the error estimates used.

Reviewer #2: This analysis included participants with and without persistent fatigue and difficulty concentrating. Using a data-driven approach, the manuscript noted heterogeneity, including symptoms such as PEM and sleep problems.

More details are necessary regarding inclusion criteria. As participants experienced persistent fatigue and difficulty concentrating, and these factors also appeared in the various factors examined. How are you distinguishing between inclusion criteria and outcomes of interest? For example, it's not surprising that the FES or DSQ would be highly correlated with being the PASC group.

The methods section notes that "only participants with complete clinical, somatic, behavioural, and cognitive data were

included." How many participants were excluded because of this criteria? What pattern of missing observations was seen in the data? At minimum, a flowchart is necessary to describe included participants. But, could other methods have been used to include these participants? Also, on this topic, the methods section later states that imputation was used. It is unclear why 1) imputation is needed if only complete data was used, and 2) why imputation could not have been used all along with whatever values were missing? How was the number of imputations selected?

This analysis has a relatively small sample to explore a large number of factors. Were there any considerations related to sample size?

The approach using factor loadings and Eigenvalues along with a Varimax rotation loses the original scale of the included factors. How should the results be interpreted? For example, in Figure 1A, how should the Eigenvalues and values along the X-axis be interpreted? And in Figure 1B, what does it mean that component 1 has a factor loading around -0.7 for the PASC group and 0.95 for the no PASC group? In Figure 2A, what do 1, 2 and 3 mean on the X-axis?

What is meant exactly by "latent factors"?

Number of comorbidities was included as a factor. It is however possible that certain comorbidities could be more relevant in some clusters than others?

Dutch is nationality, rather than ethnicity.

There is no data availability statement in the manuscript (despite a statement to the contrary on the first pages). Please include this information, as described in the PLOS Data policy.

**********

what does this mean?). If published, this will include your full peer review and any attached files.

If you choose “no”, your identity will remain anonymous but your review may still be made public.

**Do you want your identity to be public for this peer review?**  For information about this choice, including consent withdrawal, please see our Privacy Policy

Reviewer #1: No

Reviewer #2: **Yes:**  Sarah Haile

**********

[NOTE: If reviewer comments were submitted as an attachment file, they will be attached to this email and accessible via the submission site. Please log into your account, locate the manuscript record, and check for the action link "View Attachments". If this link does not appear, there are no attachment files.]

To ensure your figures meet our technical requirements, please review our figure guidelines: https://journals.plos.org/plosone/s/figures

You may also use PLOS’s free figure tool, NAAS, to help you prepare publication quality figures: https://journals.plos.org/plosone/s/figures#loc-tools-for-figure-preparation.

NAAS will assess whether your figures meet our technical requirements by comparing each figure against our figure specifications.

# Author comment: Author response to Decision Letter 1

**DOI:** 10.1371/journal.pone.0359844.r002

6 Jul 2026

Manuscript: PONE-D-25-53636

“Post-acute sequelae of COVID-19 (PASC) are associated with distinct clinical phenotypes two years after SARS-CoV-2 infection: a data-driven approach”

Dear Dr. Kassegne,

We thank you and the two reviewers for your careful and constructive evaluation of our manuscript. We have revised the manuscript point-by-point in accordance with the comments raised, as detailed below. Reviewer and editor comments are reproduced in italics, followed by our response. All changes have been made in the revised manuscript and tracked-changes version.

We hope that the revised manuscript and our responses adequately address all concerns raised.

Yours sincerely,

On behalf of all authors,

Sander C.J. Verfaillie

Editorial comments:

Comments 1-4: We revised the manuscript with respect to PLOS ONE’s style requirements, with a validated ORCID ID for the corresponding author and Competing Interest statement in standard wording, and placed an ethics statement only in the Methods section, and included captions for our supporting information files at the end of our manuscript and update in-text citations accordingly.

Comment 5. When completing the data availability statement of the submission form, you indicated that you will make your data available on acceptance. Please revise your statement, as your entire data will need to be made freely accessible if your manuscript is accepted for publication.

We furthermore agree to make the data available and have inserted the following Data Availability Statement in the manuscript. Because legal or ethical restrictions prohibit public sharing of a data set, we indicated how others may obtain access to the data.

“The Vecosco data are freely available to investigators under the conditions of a data sharing agreement. This will include group- and individual-level fully anonymized data. Requests for data should be made to prof.dr. H. Knoop.”

Comment 6. We note that you have included the phrase “data not shown” in your manuscript. PLOS does not permit references to inaccessible data.

We agree and have removed this phrase from the manuscript (page 9)

Reviewer 1:

Conceptual clarity and inferential coherence of the PCA-based analyses Because the PCA underpins the main Results and much of the Discussion, the following issues substantially affect the interpretability of the study’s core findings.

More broadly, there is a tension between the analytical method used (PCA) and the conceptual framing of the results. The Discussion repeatedly refers to latent factors, underlying constructs, and potential mechanistic or modifiable targets, which are assumptions inherent to factor-analytic or latent-variable models (e.g., EFA/CFA/SEM), but not to PCA. If the authors’ intention is to make claims about latent constructs underlying observed symptoms, a formal factor-analytic framework would be more appropriate. Alternatively, the Discussion should be revised to avoid interpretations that exceed what PCA can support.

1. Our apologies for the confusion. We performed a PCA and corrected the terminology in the Abstract, Methods, Results and Discussion sections. Please see our more detailed response to the specific points below.

TERMINOLOGY - Throughout the manuscript, the authors use closely related but not equivalent terminology interchangeably, which introduces important conceptual and methodological confusion between principal component analysis (PCA) and factor-analytic concepts. For example, the Methods state that “a PCA (…) was performed to derive data-driven components of PASC characteristics,” but shortly thereafter refer to “factor loadings” extracted after Varimax rotation and later describe the regression analyses as investigating associations with “specific latent factors.” PCA does not estimate latent variables and does not model measurement error or communalities; rather, it produces components, component loadings, and component scores as results of a variance-decomposition procedure. Terms such as “latent factors,” “factor loadings,” and “factor structure” are specific to factor-analytic models and are not interchangeable with PCA terminology.

2. We thank the reviewer for raising this important point, and we apologize for the confusion caused by our imprecise use of terminology. We performed a PCA, and we have therefore revised the manuscript throughout (Abstract, Introduction, Methods, Results, Discussion, figures and tables) to consistently use PCA-appropriate terminology. Specifically, we have changed “latent factors” to “symptom clusters” (or “components,” as appropriate), “factor loadings” to “component loadings,” and have removed language implying latent constructs. The interpretation in the Discussion has been adjusted accordingly to reflect the descriptive, variance-decomposition nature of PCA rather than a latent-variable interpretation.

COMPONENT RETENTION - Component retention appears to be based on the eigenvalue > 1 criterion. As this rule is known to be sensitive to the number of variables and to frequently overestimate the number of components, particularly in exploratory settings, the authors are encouraged to report additional retention diagnostics (e.g., scree plot inspection or parallel analysis) or to justify the robustness of the selected solution.

3. We agree and have changed figure 1A, which includes a screeplot, to enhance interpretation. We furthermore provided eigenvalues of each components with corresponding percentages of explained variance in the manuscript, to support the component retention decision, and we have reported this in the Methods/Results (page 5 and 8) and figure 1A.

LOADINGS / SCORES CONFUSION - The manuscript states that linear regression was performed “on factor loadings,” which is methodologically incorrect. Component (or factor) loadings are fixed properties of the PCA solution at the variable level and do not vary across individuals; as such, they cannot serve as dependent variables in regression analyses. The analyses appear instead to use component scores, which are subject-level quantities and are appropriate outcomes for regression. This distinction should be explicitly clarified and corrected throughout the manuscript.

4. We agree and thank the reviewer for catching this error. We confirm that the regression analyses used component scores, not component loadings. We have corrected this terminology throughout the manuscript (Methods and Results section) to consistently and accurately distinguish loadings (variable-level) from scores (subject-level).

CORRELATION VS CONFOUNDING - The manuscript reports Spearman correlations between sociodemographic variables (age, sex, education, ethnicity) and the PCA-derived components and describes this step as an assessment of “confounding effects.” However, confounding was already addressed in the subsequent multivariable regression models that adjusted for these same variables. Bivariate correlation does not assess confounding and instead only describes unadjusted associations. This analysis should therefore be described as exploratory or descriptive, or omitted.

5. We agree and have rephrased this sentence (page 5 and 6) and to describe these bivariate correlations as exploratory/descriptive associations rather than as an assessment of confounding.

INFERENTIAL TARGET / PC1 INEVITABILITY - More broadly, the objective and interpretability of the PCA-based regression analyses are unclear. Because the first principal component is, by definition, the linear combination of variables that maximizes explained variance, it is largely expected a priori that this component will show the strongest association with PASC status if group-related symptom burden constitutes a dominant source of variability. The subsequent finding that only the first unsupervised component distinguishes groups is therefore unsurprising and risks being over-interpreted as an inferential result rather than a descriptive property of the variance structure.

6. We thank the reviewer for this insightful comment. We agree with this concern and have revised the Discussion (page 12) to explicitly acknowledge this limitation and to temper the interpretation of the first principal component.

A clearer alignment between the research question and the analytical strategy would be achieved by either modeling PASC status directly—using PCA or factor scores purely as predictors in a logistic regression framework adjusted for covariates—or by testing multivariate group differences using PERMANOVA applied to the original symptom and test variables. In the latter case, an appropriate distance metric and scaling for the mixed variable types would allow formal inference on group differences in the full multivariate space, with a corresponding constrained ordination (e.g., redundancy analysis) providing an interpretable visualization. While PCA remains useful for descriptive dimension reduction and exploratory purposes (e.g., clustering), treating PCA components themselves as inferential outcomes obscures the group-comparison objective and risks over-interpreting variance-maximizing components.

7. We thank the reviewer for this suggestion. In the revised version of the manuscript we ran a logistic regression with PCA scores as predictor and PASC as dependent variable, while adjusting for covariates. In the revised version we now present Odds ratios and 95% confidence intervals (page 6 and 10).

Minor comment: choice of visualization in Figure 2B Figure 2B presents cluster distributions using pie charts. With only three groups, this visualization offers limited interpretive value and makes quantitative comparison difficult. The same information could be more clearly conveyed using a simple table, a bar plot with proportions, or even brief textual reporting of frequencies and percentages. Replacing the pie charts with a more straightforward representation would improve clarity and readability.

8. We agree and have replaced the pie charts in Figure 2B with a simple table reporting frequencies and percentages (table 3, page 11) for clearer quantitative comparison.

Minor comment: clarity of Figure 2D. Figure 2D is intended to illustrate “muscle strength and physical performance,” but the units of measurement and the nature of the displayed uncertainty are not specified in the figure keys or caption. It is therefore unclear whether the panels show group means with error intervals (and, if so, whether these represent standard deviations, standard errors, or confidence intervals) or another summary measure.

For group-level summaries with associated uncertainty, alternative visualizations such as point–range plots (means with confidence intervals), boxplots, or violin plots would improve clarity and interpretability and allow readers to better assess the distribution of the underlying data. At a minimum, the figure caption and legend should explicitly state the scale, units, and definition of the error estimates used.

9. In the revised manuscript we now provided information about the units of measurement and definition of the error in the legend. In both tests muscle strength and physical performance are assessed by a clinician-scientist and graded on a validated scale.

Reviewer #2: This analysis included participants with and without persistent fatigue and difficulty concentrating. Using a data-driven approach, the manuscript noted heterogeneity, including symptoms such as PEM and sleep problems.

More details are necessary regarding inclusion criteria. As participants experienced persistent fatigue and difficulty concentrating, and these factors also appeared in the various factors examined. How are you distinguishing between inclusion criteria and outcomes of interest? For example, it's not surprising that the FES or DSQ would be highly correlated with being the PASC group.

10. We agree that there is a substantial overlap between our inclusion criteria and variables that we examined. One explanation is that by design our aim was to investigate factors associated with PASC and we therefore selected PASC-sensitive tests. It is therefore not surprising that these highly correlated. Our inclusion criteria are based upon validated cut-offs (control versus patients) for substantial chronic fatigue and concentration problems which have also been applied in other syndromes/ diseases (e.g. ME/CFS, cancer). Notwithstanding to assess whether our inclusion criteria influenced we repeated our primary analyses with a more general Post-COVID Functional Scale (PCFS, continuous scale) and the results remained essentially unchanged (page 10). In another sensitivity analyses we re-ran PCA without FES and DSQ because of the high correlations between FES and DSQ and inclusion criteria. Results of these analyses indicated similar set of component, although explained somewhat less variance (3%) (page 9). Hence we feel that we that we have done justice to the data.

The methods section notes that "only participants with complete clinical, somatic, behavioural, and cognitive data were included." How many participants were excluded because of this criteria? What pattern of missing observations was seen in the data? At minimum, a flowchart is necessary to describe included participants. But, could other methods have been used to include these participants? Also, on this topic, the methods section later states that imputation was used. It is unclear why 1) imputation is needed if only complete data was used, and 2) why imputation could not have been used all along with whatever values were missing? How was the number of imputations selected? How many participants were excluded because of this criteria? What pattern of missing observations was seen in the data? At minimum, a flowchart is necessary …

11. In the total study we had data of n=230 individuals. Seventy-seven participants were excluded because they had missing data. Please also see response 12. We have compared demographics, fatigue and concentration severity of the complete data set with the one used for the present study and we did not see specific patterns of observations or evidence for attrition. Comparisons are presented in the supplemental data. In short, demographic and basic clinical data was comparable and there was no indication for a pattern of missing data.

To test if the missing data may give other findings we also performed imputations (n=5) on the entire dataset. PCA’s delivered essentially comparable factor loadings.

We did not use imputations all along because we found that many cases had substantial amount of missing data (2-9%) and preferred to work with the original data and subsequently test its robustness on imputed data.

In the revised manuscript we now provided the reader with the percentages of missing data for each variable, because we find this more informative than a flowchart (page 4).

It is unclear why 1) imputation is needed if only complete data was used, and 2) why imputation could not have been used all along with whatever values were missing? How was the number of imputations selected?

12. Apologies for the confusion, the primary analyses was performed on complete data (n=153). We have not used imputation all along because in some cases there was substantial amount of missing data. In addition, there are still many unknown factors that contribute to the clinical characteristics of PASC and we preferred to avoid influences to the data. However, to reassure that our results were not dependent of the cases with only available data, we repeated primary analyses with imputed data (default n=5). We have clarified the Methods to explain the rationale for using imputation in this context and how the number of imputations was selected.

This analysis has a relatively small sample to explore a large number of factors. Were there any considerations related to sample size?

13. We included all participants who have participated in the VeCosCO study, which is a prospective case-control study (Verveen et al., https://doi.org/10.1136/bmjopen-2023-072611). We agree that we examined a relatively sample size in relation to a large number of factors. We acknowledged this as a limitation in the Discussion (page 14).

The approach using factor loadings and Eigenvalues along with a Varimax rotation loses the original scale of the included factors. How should the results be interpreted? For example, in Figure 1A, how should the Eigenvalues and values along the X-axis be interpreted? And in Figure 1B, what does it mean that component 1 has a factor loading around -0.7 for the PASC group and 0.95 for the no PASC group? In Figure 2A, what do 1, 2 and 3 mean on the X-axis? What is meant exactly by "latent factors"?

14. We apologize for the confusion with respect to statistics. As noted above, we have used a PCA, and have therefore changed the formulation and interpretation throughout the manuscript from “latent factors” to “symptom clusters” (and component loadings/scores, as appropriate). We have also clarified in the Methods (page 5 and 6) and figure legends how the eigenvalues, component loadings, and component scores should be interpreted. In short, these are standardized scores (mean~0 and SD~1).

We furthermore provided information on the X-axis about the meaning of 1, 2 and 3 in Figure 2A. These represent K-means derived Cluster Numbers.

Number of comorbidities was included as a factor. It is however possible that certain comorbidities could be more relevant in some clusters than others?

15. We agree this is an interesting consideration. During the data collection we have not separately specified each comorbidity, so we are unable to investigate these effects unfortunately. We have added a comment on this point in the Discussion as a limitation (page 14)

Dutch is nationality, rather than ethnicity.

16. We have corrected this terminology throughout the manuscript.

There is no data availability statement in the manuscript (despite a statement to the contrary on the first pages). Please include this information.

17. We agree to make the data available and have inserted the following Data Availability Statement in the manuscript:

“The Vecocsco data are available to investigators under the conditions of a data sharing agreement. This will include group- and individual-level fully anonymized data. Requests for data should be made to prof.dr. H. Knoop"

Attachment Submitted filename: Response_to_reviewers.docx

# Peer review: Decision Letter 1

**DOI:** 10.1371/journal.pone.0359844.r003

**Authors:** Kokouvi Kassegne

21 Aug 2026

Dear Dr. Verfaillie,

Thank you for submitting your manuscript to PLOS One. After careful consideration, we feel that it has merit but does not fully meet PLOS One’s publication criteria as it currently stands. Therefore, we invite you to submit a revised version of the manuscript that addresses the points raised during the review process.

Please submit your revised manuscript by Oct 05 2026 11:59PM. If you will need more time than this to complete your revisions, please reply to this message or contact the journal office at plosone@plos.org. When you're ready to submit your revision, log on to https://www.editorialmanager.com/pone/ and select the 'Submissions Needing Revision' folder to locate your manuscript file.

- A letter that responds to each point raised by the academic editor and reviewer(s). You should upload this letter as a separate file labeled 'Response to Reviewers'.
- A marked-up copy of your manuscript that highlights changes made to the original version. You should upload this as a separate file labeled 'Revised Manuscript with Track Changes'.
- An unmarked version of your revised paper without tracked changes. You should upload this as a separate file labeled 'Manuscript'.

If you would like to make changes to your financial disclosure, please include your updated statement in your cover letter. Guidelines for resubmitting your figure files are available below the reviewer comments at the end of this letter.

If applicable, we recommend that you deposit your laboratory protocols in protocols.io to enhance the reproducibility of your results. Protocols.io assigns your protocol its own identifier (DOI) so that it can be cited independently in the future. For instructions see: https://journals.plos.org/plosone/s/submission-guidelines#loc-laboratory-protocols. Additionally, PLOS One offers an option for publishing peer-reviewed Lab Protocol articles, which describe protocols hosted on protocols.io. Read more information on sharing protocols at https://plos.org/protocols?utm_medium=editorial-email&utm_source=authorletters&utm_campaign=protocols.

As the corresponding author, your ORCID iD is verified in the submission system and will appear in the published article. PLOS supports the use of ORCID, and we encourage all coauthors to register for an ORCID iD and use it as well. Please encourage your coauthors to verify their ORCID iD within the submission system before final acceptance, as unverified ORCID iDs will not appear in the published article. *Only*  the individual author can complete the verification step; PLOS staff *cannot*  verify ORCID iDs on behalf of authors.

We look forward to receiving your revised manuscript.

Kind regards,

Kokouvi Kassegne

Academic Editor

PLOS One

Journal Requirements:

If the reviewer comments include a recommendation to cite specific previously published works, please review and evaluate these publications to determine whether they are relevant and should be cited. There is no requirement to cite these works unless the editor has indicated otherwise.

Please review your reference list to ensure that it is complete and correct. If you have cited papers that have been retracted, please include the rationale for doing so in the manuscript text, or remove these references and replace them with relevant current references. Any changes to the reference list should be mentioned in the rebuttal letter that accompanies your revised manuscript. If you need to cite a retracted article, indicate the article’s retracted status in the References list and also include a citation and full reference for the retraction notice.

Additional Editor Comments:

Substantial minor revisions required. Please provide a point-by-point response to the reviewer's comments and revise the manuscript accordingly.

[Note: HTML markup is below. Please do not edit.]

Reviewers' comments:

Reviewer's Responses to Questions

**Comments to the Author**

Reviewer #2: All comments have been addressed

Reviewer #3: All comments have been addressed

**********

2. Is the manuscript technically sound, and do the data support the conclusions?

Reviewer #2: (No Response)

Reviewer #3: Yes

**********

3. Has the statistical analysis been performed appropriately and rigorously?

Reviewer #2: (No Response)

Reviewer #3: Yes

**********

4. Have the authors made all data underlying the findings in their manuscript fully available?

The PLOS Data policy

Reviewer #2: (No Response)

Reviewer #3: Yes

**********

5. Is the manuscript presented in an intelligible fashion and written in standard English?

Reviewer #2: (No Response)

Reviewer #3: No

**********

Reviewer #2: (No Response)

Reviewer #3: The authors have addressed the reviewers' comments comprehensively and have substantially improved the manuscript. The statistical methodology is now much clearer, particularly regarding the appropriate interpretation of the PCA, the distinction between component loadings and component scores, and the revised regression analyses. The additional sensitivity analyses and the revised discussion strengthen the robustness and transparency of the findings.

The manuscript is well structured, the research question is clinically relevant, and the identification of different clinical phenotypes in PASC represents a valuable contribution to the field. The authors have also improved the presentation of the results by revising figures and tables, clarifying methodological details, and appropriately tempering conclusions where necessary.

I only have a few minor suggestions before publication:

1. A final proofreading for grammar and language would further improve readability, as a few minor stylistic issues remain.

2. Please ensure that PCA terminology (components, component loadings, component scores) is used consistently throughout the final manuscript.

3. Verify that all figure legends are fully self-contained, including definitions of abbreviations and statistical measures.

Overall, I believe the manuscript has been considerably strengthened during revision and is now suitable for publication after these minor editorial revisions.

**********

what does this mean?). If published, this will include your full peer review and any attached files.

If you choose “no”, your identity will remain anonymous but your review may still be made public.

**Do you want your identity to be public for this peer review?**  For information about this choice, including consent withdrawal, please see our Privacy Policy

Reviewer #2: No

Reviewer #3: **Yes:**  Jana Schellenberg

**********

[NOTE: If reviewer comments were submitted as an attachment file, they will be attached to this email and accessible via the submission site. Please log into your account, locate the manuscript record, and check for the action link "View Attachments". If this link does not appear, there are no attachment files.]

To ensure your figures meet our technical requirements, please review our figure guidelines: https://journals.plos.org/plosone/s/figures

You may also use PLOS’s free figure tool, NAAS, to help you prepare publication quality figures: https://journals.plos.org/plosone/s/figures#loc-tools-for-figure-preparation.

NAAS will assess whether your figures meet our technical requirements by comparing each figure against our figure specifications.

# Author comment: Author response to Decision Letter 2

**DOI:** 10.1371/journal.pone.0359844.r004

27 Aug 2026

Dear Dr. Kassegne,

We thank the reviewers for their careful and constructive evaluation of our manuscript in this second round. We are pleased to hear that they found the manuscript considerably strengthened during revision and now suitable for publication, pending minor editorial revisions. We have addressed all comments point-by-point, as detailed below. Reviewer comments are reproduced in italics, followed by our responses. All changes are reflected in the revised manuscript and the tracked-changes version.

We hope that the revised manuscript is suitable for publication.

Yours sincerely, on behalf of all authors,

Sander C.J. Verfaillie

Reviewer #3: The authors have addressed the reviewers' comments comprehensively and have substantially improved the manuscript. The statistical methodology is now much clearer, particularly regarding the appropriate interpretation of the PCA, the distinction between component loadings and component scores, and the revised regression analyses. The additional sensitivity analyses and the revised discussion strengthen the robustness and transparency of the findings.

The manuscript is well structured, the research question is clinically relevant, and the identification of different clinical phenotypes in PASC represents a valuable contribution to the field. The authors have also improved the presentation of the results by revising figures and tables, clarifying methodological details, and appropriately tempering conclusions where necessary.

I only have a few minor suggestions before publication:

1. A final proofreading for grammar and language would further improve readability, as a few minor stylistic issues remain.

We have corrected grammar and language issues in the revised manuscript.

2. Please ensure that PCA terminology (components, component loadings, component scores) is used consistently throughout the final manuscript.

We have made several corrections to ensure consistent PCA terminology in the revised manuscript.

3. Verify that all figure legends are fully self-contained, including definitions of abbreviations and statistical measures.

We have revised the figure legends so that these are fully self-contained, including definitions of abbreviations.

Attachment Submitted filename: Rebuttal.docx

# Peer review: Decision Letter 2

**DOI:** 10.1371/journal.pone.0359844.r005

**Authors:** Kokouvi Kassegne

20 Sep 2026

Post-acute sequelae of COVID-19 (PASC) are associated with distinct clinical phenotypes two years after SARS-CoV-2 infection: A data-driven approach

PONE-D-25-53636R2

Dear Dr. Sander C.J. Verfaillie,

We’re pleased to inform you that your manuscript has been judged scientifically suitable for publication and will be formally accepted for publication once it meets all outstanding technical requirements.

Within one week, you’ll receive an e-mail detailing the required amendments. When these have been addressed, you’ll receive a formal acceptance letter and your manuscript will be scheduled for publication.

An invoice will be generated when your article is formally accepted. Please note, if your institution has a publishing partnership with PLOS and your article meets the relevant criteria, all or part of your publication costs will be covered. Please make sure your user information is up-to-date by logging into Editorial Manager at Editorial Manager® and clicking the ‘Update My Information' link at the top of the page. For questions related to billing, please contact billing support.

If your institution or institutions have a press office, please notify them about your upcoming paper to help maximize its impact. If they’ll be preparing press materials, please inform our press team as soon as possible -- no later than 48 hours after receiving the formal acceptance. Your manuscript will remain under strict press embargo until 2 pm Eastern Time on the date of publication. For more information, please contact onepress@plos.org.

Kind regards,

Kokouvi Kassegne

Academic Editor

PLOS One

Additional Editor Comments (optional):

Accept for publication

Reviewers' comments:

Reviewer's Responses to Questions

**Comments to the Author**

Reviewer #3: All comments have been addressed

**********

2. Is the manuscript technically sound, and do the data support the conclusions?

Reviewer #3: Partly

**********

3. Has the statistical analysis been performed appropriately and rigorously?

Reviewer #3: Yes

**********

4. Have the authors made all data underlying the findings in their manuscript fully available?

The PLOS Data policy

Reviewer #3: Yes

**********

5. Is the manuscript presented in an intelligible fashion and written in standard English?

Reviewer #3: (No Response)

**********

Reviewer #3: The authors have adequately addressed my previous comments, and the manuscript has been further improved. The remaining issues are minor editorial inconsistencies that do not affect the overall interpretation or scientific conclusions of the study. I therefore recommend acceptance.

I would only encourage the authors to correct the remaining minor terminology and language inconsistencies during final proofreading, particularly the occasional use of “factor loadings” instead of “component loadings” and “linear regression” instead of “logistic regression” in the Abstract.

**********

what does this mean?). If published, this will include your full peer review and any attached files.

If you choose “no”, your identity will remain anonymous but your review may still be made public.

**Do you want your identity to be public for this peer review?**  For information about this choice, including consent withdrawal, please see our Privacy Policy

Reviewer #3: **Yes:**  PD Dr. med. Jana Schellenberg

**********

# Editor report: Acceptance letter

**DOI:** 10.1371/journal.pone.0359844.r006

**Authors:** Kokouvi Kassegne

PONE-D-25-53636R2

PLOS One

Dear Dr. Verfaillie,

I'm pleased to inform you that your manuscript has been deemed suitable for publication in PLOS One. Congratulations! Your manuscript is now being handed over to our production team.

At this stage, our production department will prepare your paper for publication. This includes ensuring the following:

* All references, tables, and figures are properly cited

* All relevant supporting information is included in the manuscript submission,

* There are no issues that prevent the paper from being properly typeset

You will receive further instructions from the production team, including instructions on how to review your proof when it is ready. Please keep in mind that we are working through a large volume of accepted articles, so please give us a few days to review your paper and let you know the next and final steps.

Lastly, if your institution or institutions have a press office, please let them know about your upcoming paper now to help maximize its impact. If they'll be preparing press materials, please inform our press team within the next 48 hours. Your manuscript will remain under strict press embargo until 2 pm Eastern Time on the date of publication. For more information, please contact onepress@plos.org.

You will receive an invoice from PLOS for your publication fee after your manuscript has reached the completed accept phase. If you receive an email requesting payment before acceptance or for any other service, this may be a phishing scheme. Learn how to identify phishing emails and protect your accounts at https://explore.plos.org/phishing.

If we can help with anything else, please email us at customercare@plos.org.

Thank you for submitting your work to PLOS One and supporting open access.

Kind regards,

PLOS One Editorial Office Staff

on behalf of

Dr. Kokouvi Kassegne

Academic Editor

PLOS One
